# Association of Neighborhood-Level Disadvantage With Neurofibrillary Tangles on Neuropathological Tissue Assessment

**DOI:** 10.1001/jamanetworkopen.2022.8966

**Published:** 2022-04-28

**Authors:** W. Ryan Powell, Megan Zuelsdorff, Sarah A. Keller, Tobey J. Betthauser, Robert A. Rissman, Barbara B. Bendlin, Amy J. H. Kind

**Affiliations:** 1 Center for Health Disparities Research, University of Wisconsin School of Medicine and Public Health, Madison; 2 Wisconsin Alzheimer’s Disease Research Center, University of Wisconsin School of Medicine and Public Health, Madison; 3 University of Wisconsin School of Nursing, Madison; 4 Department of Medicine, Geriatrics Division, University of Wisconsin School of Medicine and Public Health, Madison; 5 Department of Neurosciences, University of California, San Diego; 6 Shiley-Marcos Alzheimer's Disease Research Center, University of California, San Diego, La Jolla; 7 Wisconsin Alzheimer’s Institute, University of Wisconsin School of Medicine and Public Health, Madison; 8 Geriatric Research Education and Clinical Center, William S. Middleton Hospital, Department of Veterans Affairs, Madison, Wisconsin

## Abstract

This cross-sectional study evaluates income, educational level, employment status, and neighborhood and their association with brain changes in decedents with Alzheimer disease and related dementias.

## Introduction

The social exposome measures all of the social exposures that a person experiences over a lifetime. Researchers are only beginning to understand the role of upstream, neighborhood-level factors in Alzheimer disease and related dementias (ADRD) risk and their association with biological pathways affecting ADRD.

Neighborhood disadvantage, a social exposome measure reflecting income, educational level, employment status, and housing in a Census-block group or neighborhood, has been associated with markers of ADRD brain health, including amyloid plaque.^1^ Whether this association extends to neurofibrillary pathology is unknown. This study evaluated the association between neurofibrillary tangles and neighborhood disadvantage.

## Methods

This cross-sectional study was conducted using a sample of decedents from 2 Alzheimer’s Disease Research Center (ADRC) brain banks with previously assessed neurofibrillary tangle deposition and neighborhood disadvantage ranking from 1993 to 2016. Before death, decedents were recruited by ADRC brain donor programs and consented to brain donation for research purposes. The institutional review boards of University of Wisconsin and University of California, San Diego exempted the study because it was not human participant research. We followed the STROBE reporting guideline.

We abstracted neurofibrillary tangle B scores, per National Institute on Aging and Alzheimer’s Association neuropathological change guidelines,^2^ from the standardized Neuropathology Data Set form or original autopsy reports (following established methods and neuropathologist guidance from both ADRCs) to measure Alzheimer disease–associated neurofibrillary pathology.^1,3^ Twenty-five decedents without neurofibrillary tangle assessment were excluded. Decedents’ last address was geolinked to their statewide ranking of neighborhood disadvantage using a time-concordant area deprivation index, with higher values denoting greater neighborhood disadvantage.^4^ Generalized ordered logistic regression with site-level clustered SEs was used to model the ordinal B score adjusted for covariates regularly available across the 24-year data time frame: age, sex, and year of death of 2005 or later (ie, introductory year for standard reporting using the Uniform Data Set). Data were analyzed from May 3 to November 17, 2021, using Stata/MP version 16.1 (StataCorp LLC).

## Results

The sample of 428 decedents had a mean (SD) age of 80.5 (9.1) years (237 men [55.4%] and 191 women [44.6%]) and tended to be from less disadvantaged neighborhoods (mean [SD] area deprivation index decile rank: 3.8 [2.4]) (Table). Nearly all (95.8%) had neurofibrillary tangles, which was consistent with ADRC brain donation samples. Modeled analysis suggested that, for every decile increase in neighborhood disadvantage, there was a 5% increase in the odds of a higher B score (odds ratio [OR], 1.05; 95% CI, 1.01-1.08) after adjustments (Table). This translated into an estimated 56% increased odds of a higher B score for those in the most disadvantaged neighborhood decile (OR, 1.56; 95% CI, 1.09-2.23) (Figure). Sensitivity analysis using Braak staging, instead of B scores, suggested similar estimated odds (OR, 1.04; 95% CI, 1.02-1.06).

**Table.** Decedent Sample Characteristics and Adjusted Odds of a Higher Neurofibrillary Tangle B Score

Characteristic	Participants, No. (%) (N = 428)	Adjusted OR (95% CI)^a^		
		B score 1, 2, 3 vs 0	B score 2, 3 vs 0, 1	B score 3 vs 0, 1, 2
Neurofibrillary tangle B score^b^				
0: No neurofibrillary tangles	18 (4.2)	NA	NA	NA
Braak stage				
1: I and II	92 (21.5)	NA	NA	NA
2: III and IV	79 (18.5)	NA	NA	NA
3: V and VI	239 (55.8)	NA	NA	NA
Neighborhood disadvantage, mean (SD)^c^^,^^d^	3.8 (2.4)	1.05 (1.01-1.08)	1.05 (1.01-1.08)	1.05 (1.01-1.08)
Age group, y				
<70	62 (14.5)	1 [Reference]	1 [Reference]	1 [Reference]
70-79^d^	110 (25.7)	1.73 (1.60-1.86)	1.73 (1.60-1.86)	1.73 (1.60-1.86)
80-89^d^	189 (44.2)	1.27 (1.05-1.54)	1.27 (1.05-1.54)	1.27 (1.05-1.54)
≥90^e^	67 (15.7)	4.87 (2.24-10.63)	1.09 (1.00-1.18)	0.71 (0.51-1.01)
Female sex	191 (44.6)	1 [Reference]	1 [Reference]	1 [Reference]
Male sex^e^	237 (55.4)	1.19 (0.86-1.63)	0.58 (0.57-0.60)	0.67 (0.49-0.90)
Year of death 2005 and later^e^	342 (79.9)	1.14 (0.83-1.55)	0.66 (0.40-1.07)	0.65 (0.55-0.77)

Abbreviations: NA, not applicable (because variable was the modeled outcome); OR, odds ratio.

^a^ Adjusted ORs (95% CI) were calculated using generalized ordered logistic regression. Adjusted model included all variables listed in the table. Twenty-five decedents were excluded who, when compared with those who were included, had no neurofibrillary tangle information, were younger (mean [SD] age, 72.5 [14.7] years vs 80.5 [9.1] years; *P* = .01), and died in 2005 or later (96.0% vs 79.9%; χ^2^ test of independence *P* = .047).

^b^ B score categorizes the deposition of tangles within certain brain regions in 4 stages: B0 (neurofibrillary tangles not present), B1 (Braak stages I and II; transentorhinal stages), B2 (Braak stages III and IV; limbic stages), and B3 (Braak stages V and VI; isocortical stages).

^c^ Statewide decile ranking reflected income, educational level, employment status, and housing neighborhood dimensions. Higher values denoted greater levels of neighborhood disadvantage. All deciles were represented in the study (area deprivation index range 1-10): decile 1 = 80 (18.7%), decile 2 = 71 (16.6%), decile 3 = 64 (15.0%), decile 4 = 67 (15.7%), decile 5 = 51 (11.9%), decile 6 = 35 (8.2%), decile 7 = 24 (5.6%), decile 8 = 12 (2.8%), decile 9 = 11 (2.6%), and decile 10 = 13 (3.0%).

^d^ Estimates were the same because the proportional odds assumption was met when comparing the odds at each increasing level of the B score.

^e^ Estimates were different because the proportional odds assumption was violated when comparing the odds at each increasing level of the B score.

**Figure.**
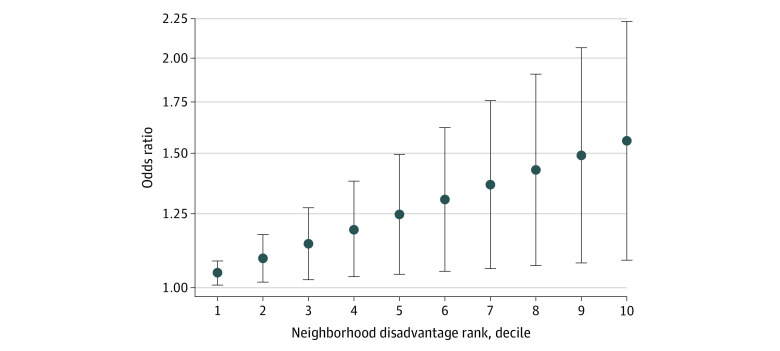
Odds of a Higher Neurofibrillary Tangle B Score by Neighborhood Disadvantage Decile Rank Error bars depict 95% CIs. Sample consisted of 428 decedents, and postestimation linear combination of model parameters are shown.

## Discussion

With these new findings, neighborhood disadvantage has now been found to be associated with neurofibrillary tangles and amyloid plaques,^1^ the primary pathological features of Alzheimer disease. Study limitations emphasize the need for additional infrastructure, data, and insight to address selection bias in brain donation, underrepresentation of decedents from disadvantaged neighborhoods, and generalizability.

Neighborhood disadvantage may serve a role in identifying ADRD biological processes and/or be a marker of related adverse exposures. Therefore, a nuanced understanding is needed of the pathways through which neighborhood conditions may associate or interact with other factors to affect ADRD-related brain changes.^5^ Mechanisms linking neighborhood disadvantage with tau accumulations might include multiple and overlapping factors (eg, stress, depression, sleep disruption, and constraints on health behaviors; pollution; and cardiovascular risks).^6^ Future work will require coordinated involvement among ADRCs for larger, more generalizable samples and additional data linkages to explore the mediating and moderating risk factors involved.

Neuropathological changes in ADRD accumulate over decades. Life-course approaches to describing neighborhood disadvantage exposure should include testing dose-response associations, identifying critical thresholds that place people at elevated risk, pinpointing sensitive life periods, and uncovering factors that mitigate the impact of exposure.
